# Relationship of acute axonal damage, Wallerian degeneration, and clinical disability in multiple sclerosis

**DOI:** 10.1186/s12974-017-0831-8

**Published:** 2017-03-17

**Authors:** Shailender Singh, Tobias Dallenga, Anne Winkler, Shanu Roemer, Brigitte Maruschak, Heike Siebert, Wolfgang Brück, Christine Stadelmann

**Affiliations:** 1 Institute of Neuropathology, University Medical Center, Göttingen, Germany; 2 Cellular Microbiology, Research Center Borstel, Borstel, Germany; 3 Department of Neurology, Rigshospitalet, Copenhagen, Denmark

**Keywords:** Multiple sclerosis, Experimental autoimmune encephalomyelitis, Wallerian degeneration, Axonal damage, *Wld*^*S*^

## Abstract

**Background:**

Axonal damage and loss substantially contribute to the incremental accumulation of clinical disability in progressive multiple sclerosis. Here, we assessed the amount of Wallerian degeneration in brain tissue of multiple sclerosis patients in relation to demyelinating lesion activity and asked whether a transient blockade of Wallerian degeneration decreases axonal loss and clinical disability in a mouse model of inflammatory demyelination.

**Methods:**

Wallerian degeneration and acute axonal damage were determined immunohistochemically in the periplaque white matter of multiple sclerosis patients with early actively demyelinating lesions, chronic active lesions, and inactive lesions. Furthermore, we studied the effects of Wallerian degeneration blockage on clinical severity, inflammatory pathology, acute axonal damage, and long-term axonal loss in experimental autoimmune encephalomyelitis using Wallerian degeneration slow (*Wld* ^*S*^) mutant mice.

**Results:**

The highest numbers of axons undergoing Wallerian degeneration were found in the perilesional white matter of multiple sclerosis patients early in the disease course and with actively demyelinating lesions. Furthermore, Wallerian degeneration was more abundant in patients harboring chronic active as compared to chronic inactive lesions. No co-localization of neuropeptide Y-Y1 receptor, a *bona fide* immunohistochemical marker of Wallerian degeneration, with amyloid precursor protein, frequently used as an indicator of acute axonal transport disturbance, was observed in human and mouse tissue, indicating distinct axon-degenerative processes. Experimentally, a delay of Wallerian degeneration, as observed in *Wld* ^*S*^ mice, did not result in a reduction of clinical disability or acute axonal damage in experimental autoimmune encephalomyelitis, further supporting that acute axonal damage as reflected by axonal transport disturbances does not share common molecular mechanisms with Wallerian degeneration. Furthermore, delaying Wallerian degeneration did not result in a net rescue of axons in late lesion stages of experimental autoimmune encephalomyelitis.

**Conclusions:**

Our data indicate that in multiple sclerosis, ongoing demyelination in focal lesions is associated with axonal degeneration in the perilesional white matter, supporting a role for focal pathology in diffuse white matter damage. Also, our results suggest that interfering with Wallerian degeneration in inflammatory demyelination does not suffice to prevent acute axonal damage and finally axonal loss.

**Electronic supplementary material:**

The online version of this article (doi:10.1186/s12974-017-0831-8) contains supplementary material, which is available to authorized users.

## Background

Axonal damage and loss are the key structural features in multiple sclerosis patients and the most important correlates of persistent disability [1–3]. Also, the insidious clinical worsening in later stages of the disease, often independent of newly formed lesions and largely non-responsive to immunomodulatory treatments, but reflected in important brain and cervical spinal atrophy, is considered due to cumulative axonal loss [4–7]. Reports on axonal loss in the normal-appearing white matter (NAWM) and spinal cord range from ∼20 to 55%, whereas in chronic multiple sclerosis lesions, axonal reduction of up to 70% has been reported [8]. Axonal degeneration can occur via several mechanisms, the most prominent being anterograde (or Wallerian) and retrograde (“dying back”) degeneration [9]. Inflammation-associated axonal transport disturbances, so-called “focal axonal degeneration,” may precede axonal transection and ensuing axonal self-destruction by Wallerian degeneration [10].

Axonal transport disturbances, as visualized by the accumulation of anterogradely transported proteins such as amyloid precursor protein (APP) and synaptophysin, are often used as indicators of “acute axonal damage,” and the density of APP-positive axonal profiles is highest in the earliest stages of multiple sclerosis lesion formation [2, 11, 12]. Axons with transport disturbance are also present at relatively high density in the rims of chronic active, smoldering lesions and may be found in the NAWM [2, 13, 14]. Whether and to which extent axonal transport disturbances are reversible or lead to definite, irreversible axonal transection, is not yet clear. Correlations of APP-positive axonal profiles with the density of macrophages have been repeatedly shown for early lesions; however, in chronic disease, also a correlation with T cells was observed [2, 14–16].

In experimental models of inflammatory demyelination, the accumulation of organelles and proteins in axons corresponds to “focal axonal degeneration,” i.e., localized axonal swelling. Importantly, a proportion of axons showing focal axonal degeneration finally undergo transection and degenerate [10]. In experimental autoimmune encephalomyelitis (EAE), the progression to axonal transection is largely prevented by treatment with ROS/NOS scavengers, and axonal morphology is restored in the majority of treated axons studied [17]. Demyelination is not a prerequisite for focal axonal degeneration, supporting the contribution of highly lipid-soluble reactive species to axonal damage [10, 17–19]. Of note, however, chronic axonal transport disturbances may lead to distal axonal “malnutrition” and dying back, highlighting the importance of early therapeutic intervention [17].

Axons proceeding from focally disturbed axonal transport to axonal transection undergo a series of orderly events, namely, an acute retraction process, both proximally and distally, termed acute axonal degeneration, followed by classical Wallerian degeneration of the distal axon part [20]. Both processes are, at least in part, mediated by similar molecular mechanisms and contribute to the important physical “gap” between the proximal and distal axon stump rendering regenerative attempts challenging. As of yet, it is not well understood when the majority of Wallerian degeneration, the final process of axonal self-destruction, takes place in multiple sclerosis. In particular, it has not been explored whether Wallerian degeneration is abundant in the chronic disease stage of the disease.

Thus, to better understand the dynamics of axonal loss in patients with multiple sclerosis, we set out to study the timing and extent of Wallerian degeneration in patients with short- and long-standing disease harboring early- and late-stage lesions. Furthermore, experimentally, we strived to determine whether delaying Wallerian degeneration helps to decrease inflammatory axonal damage in a model of multiple sclerosis. Our data indicate that axonal transection and ensuing axonal self-destruction is most pronounced in the periplaque white matter of patients with early demyelinating lesions. Of note, however, also the presence of chronic active lesions associates with a high density of neuropeptide Y-Y1 receptor (NPY-Y1R)-positive axonal profiles, underlining the notion that focal demyelinated lesions are the key contributors to axonal demise in the periplaque and normal-appearing white matter. Experimentally, we find that neither acute axonal damage nor persistent axonal loss is ameliorated in focal EAE lesions in Wallerian degeneration slow (*Wld* ^*S*^) compared to wild-type (WT) mice, indicating limited sharing of molecular mechanisms between acute axonal transport disturbance and Wallerian degeneration [21]. Our study underlines the role of focal white matter lesions for axonal loss in the NAWM of patients with multiple sclerosis and supports the concept of early pharmacological interventions to prevent highly vulnerable, transport-deficient axons showing signs of focal axonal degeneration from transection.

## Methods

### Brain tissue from patients with multiple sclerosis

We investigated paraffin-embedded archival brain tissue from 17 autopsied multiple sclerosis patients and 14 biopsied patients diagnosed with inflammatory demyelination consistent with multiple sclerosis. A total of 34 [16] tissue specimens, which included non-demyelinated white matter regions, were used for the study. All biopsy samples analyzed contained non-demyelinated periplaque white matter (PPWM) areas contiguous with early/late active (*n* = 6) or inactive (*n* = 9) lesions. For direct comparison, non-demyelinated white matter tissue adjacent to 5 active lesions, 7 chronic active, and 7 chronic inactive lesions was analyzed in multiple sclerosis autopsy tissue. All lesions fulfilled the criteria for the diagnosis of multiple sclerosis [22]. Lesional activity was determined using previously described criteria [23]. The biopsies were performed in different neurosurgical centers to exclude neoplastic or infectious diseases. Specimens were sent to the Department of Neuropathology in Göttingen, Germany, for a second opinion. The patients’ clinical characteristics are summarized in Table 1.

**Table 1:** Characteristics of multiple sclerosis patients included in the study

Sex; female *n* = 16, male *n* = 15	
Age; 47 years (median), 19–76 (range)	
Clinical diagnosis^a^	
Clinically isolated syndrome suggestive of multiple sclerosis; *n* = 9 (9 biopsies)	
Primary progressive multiple sclerosis; *n* = 6 (6 autopsies)	
Relapsing-remitting multiple sclerosis; *n* = 4 (4 biopsies)	
Secondary progressive multiple sclerosis; *n* = 9 (1 biopsy, 8 autopsies)	
Disease duration of biopsied patients^b^; 42 days (median), 9–540 days (range)	
Disease duration of autopsied patients^b^; 19 years (median), 6–34 years (range)	

^a^Clinical course was not available for three autopsy patients

^b^Time from the first symptoms to biopsy/autopsy

### Brain tissue from stroke patients

Axonal transport disturbances and Wallerian degeneration are typical and abundant after brain ischemia. To study the spatial relation of the two phenomena in a prototypic human disease, we analyzed archival paraffin-embedded brain biopsy tissue from four patients (three females, one male; median age = 54 years; range 49–63 years) with ischemic stroke lesions. Lesions were characterized by tissue necrosis as evidenced by massive axonal loss and dense macrophage infiltration. Axonal swellings in a typical ischemic pattern were abundant at the lesion borders.

### Mice

Female 8–10-week-old *Wld* ^*S*^ (C57BL/6 OlaHsd) and C57BL/6 mice were obtained from the Harlan Laboratories, UK. The *Wld* ^*S*^ mouse strain is characterized by an 85-kb tandem triplication on chromosome four that occurred as a spontaneous mutation in the B6 strain in the 1940s, leading to the expression of an Ube4b/Nmnat chimeric protein. Mutant mice do not show a spontaneous phenotype. All mice had free access to water and chow and were included in the experiments after at least 5 days of acclimatization.

### EAE induction and clinical evaluation

EAE was induced by subcutaneous injection of 200 μg myelin oligodendrocyte glycoprotein (MOG)-peptide_35–55_ emulsified in complete Freund’s adjuvant (CFA) containing 1 mg/ml inactivated *Mycobacterium tuberculosis*. Control mice were injected with CFA alone*.* Three hundred nanogram pertussis toxin was injected i.p. at day 0 and day 2 after immunization. Clinical deficits were assessed daily by a blinded observer using the following scoring system: 0=no symptoms, 0.5=partial tail paresis, 1.0=complete tail paralysis, 1.5=slight hind limb paresis, 2.0=distinct hind limb paresis, 2.5=severe hind limb paresis, 3.0=complete hind limb paralysis, 3.5=slight forelimb paresis, 4.0=tetraparesis, 4.5=moribund, and 5.0=death. Mice were euthanized when reaching a score of 3.5.

### Histopathology

At the end of the EAE experiments, animals were deeply anesthetized and perfused with phosphate buffered saline (PBS) (pH 7.4) followed by 4% paraformaldehyde (PFA) in PBS. The spinal cords (SC) were dissected, and at least eight transverse sections were embedded in paraffin. One to three micrometer-thick sections were stained with hematoxylin-eosin (HE), Luxol Fast Blue/periodic acid Schiff’s reagent (LFB/PAS), and Bielschowsky silver impregnation to determine inflammation, demyelination, and axonal loss. Immunohistochemistry (IHC) was performed using the primary antibodies listed in Table 2. For antigen retrieval, tissue slices were microwaved in 10 mM citrate buffer (pH 6.0) 3 × 5 min. Bound antibodies were visualized using an appropriate biotinylated secondary antibody and an avidin-peroxidase-DAB technique. Negative control sections were incubated without primary antibodies or with irrelevant primary antibodies of the respective isotypes. Slices were counterstained with hemalaun and cover-slipped. Double fluorescence labeling with two mouse monoclonal primary antibodies was carried out as described previously [24].

**Table 2:** Antibodies used for immunohistochemistry

Target	Antibody type (clone)	Dilution	Pre-treatment	Source
Amyloid precursor protein (APP)	Mouse mAb *(*22C11)	1:2000	Citrate MW	Chemicon International, USA
Neuropeptide Y receptor Y1 (NPY-Y1R)	Rabbit polyAb #96106	1:1000	Tris-EDTA MW	CURE/UCLA, USA
Myelin basic protein (MBP)	Rabbit polyAb	1:1000	None	DakoCytomation, Denmark
MBP peptic fragment 70–89	Mouse mAb (SMI94)	1:5000	Steam	Covance Inc., USA
Myelin proteolipid protein (PLP)	Mouse mAb (Plpc 1)	1:500	Citrate MW	Biozol, Germany
Myelin oligodendrocyte glycoprotein (MOG)	Rat polyAb	1:1000	Citrate MW	[75]
Myelin-associated glycoprotein (MAG)	Rabbit polyAb	1:10	Citrate MW	[76]
2′,3′-Cyclic-nucleotide 3′-phosphodiesterase (CNPase)	Mouse mAb (SMI91)	1:200	Citrate MW	Covance Inc., USA
Monocytes, activated microglia	Mouse mAb (KiM1P)	1:5000	Citrate MW	[77]
Early-activated macrophages (S100A9)	Rabbit polyAb	1:500	Citrate MW	[78]
Phosphorylated NFs	Mouse mAb (SMI31)	1:10000	Citrate MW	Covance Inc., USA
Non-phosphorylated NFs	Mouse mAb (SMI32)	1:1000	Citrate MW	Covance Inc., USA
Hypo-phosphorylated NFs	Mouse mAb (SMI35)	1:10000	Citrate MW	Covance Inc., USA
NF-low-molecular-weight (NF68)	Mouse mAb (NR4)	1:100	Citrate MW	Sigma Chemical Company, USA
NF-high-molecular-weight (NF200)	Mouse mAb (N52)	1:400	Citrate MW	Sigma Chemical Company, USA
Growth-associated protein 43 (GAP43)	Mouse mAb (9-1E12)	1:4000	Citrate MW	Chemicon International, USA
Synaptophysin (protein p38)	Mouse mAb (SY38)	1:10	Citrate MW	DakoCytomation, Denmark

*NF* neurofilament, *mAb* monoclonal antibody, *polyAb* polyclonal antibody, *MW* microwave pre-treatment

### Mouse sciatic nerve transection

Four female C57BL/6 mice were used to study sciatic nerve axotomy. They were deeply anesthetized by intraperitoneal injection of ketamine hydrochloride (“Ketanest Inresa,” 50 mg/ml, Inresa, Freiburg, Germany) mixed with xylazine hydrochloride (“Rompun” 2%, Bayer, Leverkusen, Germany) in a ratio of 2:1 (0.4 mg Ketanest and 2 mg Rompun for each mouse). Skin and muscles above the right femur were opened by fine scissors, and the sciatic nerve was completely transected. Subsequently, muscle and skin were closed by suture (Ethicon). The mice were kept for 6 days under a 12-h dark-light cycle and given food and water ad libitum. The animals were perfused transcardially with PBS and 4% PFA, and the sciatic nerves dissected. The contralateral nerves and a sciatic nerve from an animal without axotomy served as controls. Sciatic nerves were post-fixed in 4% PFA overnight and embedded in paraffin. Microtome sections of 1–3 μm thickness were de-paraffinized, pre-treated by cooking in citrate buffer (pH 6) for 10 min, and subjected to IHC.

### Data acquisition and analysis

Tissue sections were analyzed using an Olympus BX51 fluorescence microscope equipped with a DP71 CCD camera (Olympus Optical Co, Ltd., Hamburg, Germany), a Zeiss Cell Observer microscope with an AxioCam ICc 3 CCD camera (Carl Zeiss MicroImaging, Ltd., Göttingen, Germany), or by confocal laser scanning microscopy with a Fluoview 1000 Olympus microscope. Transverse spinal cord (SC) sections at the cervical, thoracic, lumbar, and sacral levels were used for quantitative analysis. The extent of acute axonal damage of axons undergoing Wallerian degeneration and the density of healthy phosphorylated axons was calculated by counting APP^+^, NPY-Y1R^+^, or SMI31^+^ profiles in spinal cross sections within at least five white matter lesions per mouse at ×400 magnification using an ocular counting grid. Counts are given as immunopositive axonal profiles per square millimeter. Relative axonal densities within lesions were determined in sections stained with Bielschowsky’s silver impregnation by using an axonal counting grid with 25 cross-points [25]. The number of axons intersecting with the crossing points was determined as a fraction of the total number of cross-points at a magnification of ×1000, under oil immersion. The value obtained in control animals was set to 100%. The degree of axon reduction in the lesion is given as the percentage of axon density compared with the control animals. To determine the extent of demyelination, digital images of LFB/PAS-stained SC cross sections were recorded through an Olympus light microscope with a CCD DP71 camera at ×100 magnification. Using the computer program Cell^F (Soft Imaging Systems^®^), demyelinated white matter areas were measured, and the percentage with respect to total white matter was calculated. The inflammatory index was defined as the mean number of perivascular infiltrates within the SC parenchyma of 8–10 spinal cross sections per animal. All the images were prepared in Adobe Photoshop CS4 Version 11.0.2.

### Statistical analysis

Statistical analyses were carried out using Microsoft Office Excel 2007 and GraphPad Prism (GraphPad Software, La Jolla, USA). After normality testing, an unpaired *t* test was applied to determine potential differences in the mean clinical severity scores of WT and *Wld* ^*S*^ mice at day 20 and day 40 after disease onset. Histological data were analyzed by the non-parametric Mann-Whitney *U* test; animals in two groups (i.e., *Wld* ^*S*^ and WT) were compared on the basis of closely matched clinical scores. The influence of genotype and disease stage on inflammation and demyelination was determined using a two-way-ANOVA. Statistical significance was defined as *p* < 0.05. All quantitative morphological data are expressed as mean ± standard error of the mean (SEM) in the EAE studies.

## Results

### Prevalence of Wallerian degeneration in patients with early- and late-stage multiple sclerosis

**Fig. 1:**
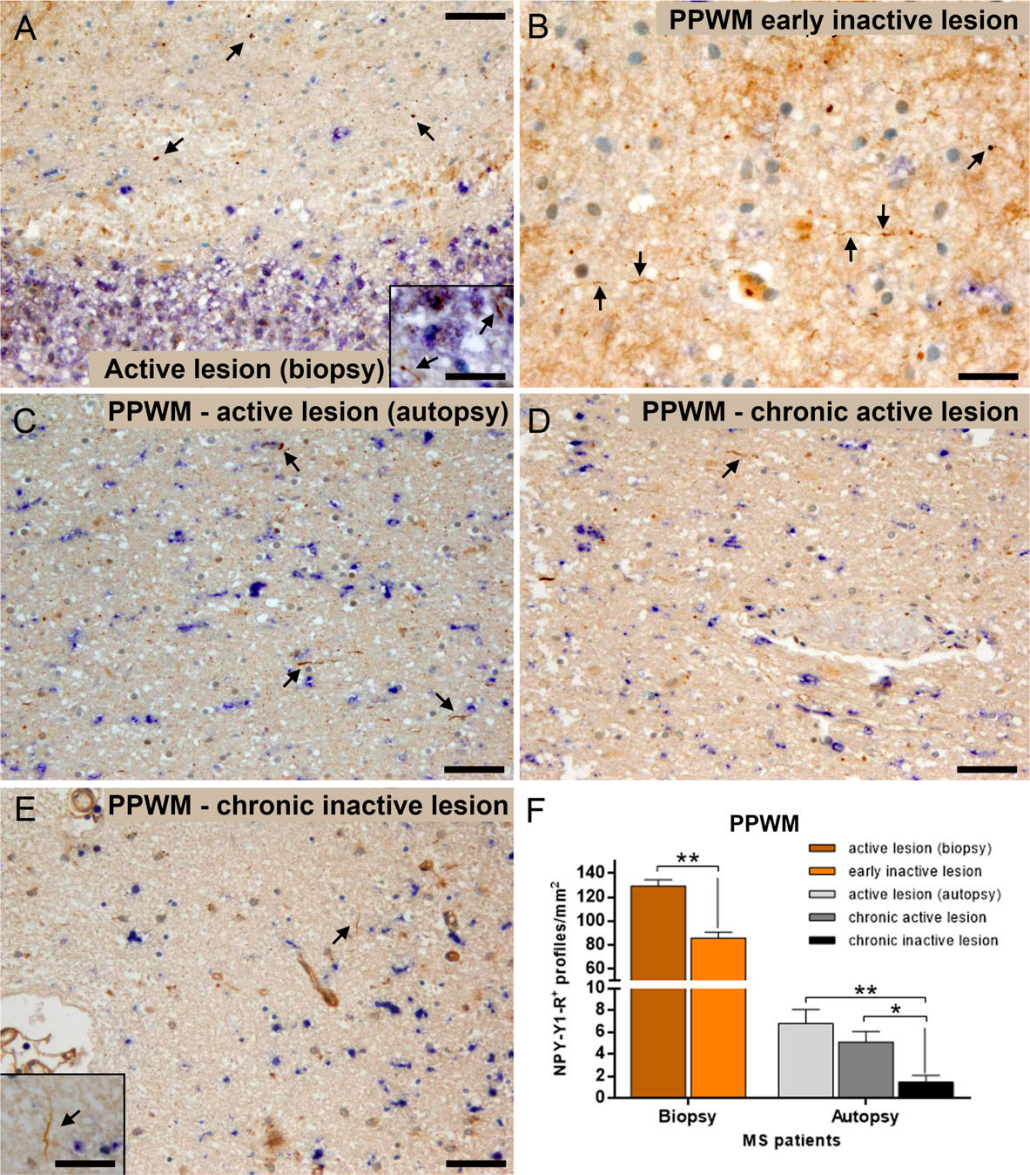
Wallerian degeneration is most frequent in the PPWM of lesions with ongoing demyelinating activity. NPY-Y1R IHC was performed on multiple sclerosis biopsy and autopsy tissue samples containing active, early inactive, chronic active, and chronic inactive lesions (**a**–**e**). Double IHC of NPY-Y1R (*red*, **a**) with the pan-macrophage marker KiM1P (*blue*, **a**) showed very little evidence for Wallerian degeneration (*arrows*) in the actively demyelinating lesion edge of a multiple sclerosis biopsy (**a**, *inset*). The density of NPY-Y1R^+^ axonal profiles indicating Wallerian degeneration was significantly higher in PPWM areas of active and chronic active lesions compared to early inactive and chronic inactive lesions in both early and chronic multiple sclerosis patients (**f**). Significantly higher numbers of NPY-Y1R^+^ axonal profiles (*arrows*) were detected in the PPWM of early multiple sclerosis patients (biopsies; **a**–**b**, **f**) compared to chronic multiple sclerosis patients (autopsies; **c**–**f**). *Error bars*=SEM, *p < 0.05, **p < 0.01, ***p < 0.001. *Scale bars*; **a** 100 μm; **b** 25 μm, **c**–**e** 50 μm; (*inset* **a**, **e**) 10 μm

Wallerian degeneration is thought to reflect a clinically relevant component of disability and disease progression in multiple sclerosis. However, the contribution of Wallerian degeneration to multiple sclerosis pathology is not yet known. Here, we studied the occurrence and extent of Wallerian degeneration in biopsy multiple sclerosis tissue from patients with short duration as well as in autopsied patients with chronic multiple sclerosis. Using NPY-Y1R immunohistochemistry, Wallerian degeneration was quantified in areas of PPWM brain tissue from 31 multiple sclerosis patients. We observed widespread occurrence of NPY-Y1R^+^ degenerating axonal fibers in non-demyelinated PPWM areas in multiple sclerosis patients (Fig. 1). Patients were divided into early and chronic disease groups according to disease duration, i.e., time from the first symptoms to biopsy/autopsy. The number of NPY-Y1R^+^ axons in multiple sclerosis PPWM was significantly higher in the early as compared to the chronic disease stage (early; 113.6 ± 7, chronic; 4.6 ± 0.8 profiles/mm^2^; ***p < 0.001). In early multiple sclerosis, the number of axons undergoing Wallerian degeneration was significantly higher in PPWM connected to active lesions as compared to early inactive lesions (PPWM-active; 129.1 ± 5, early inactive; 85.9 ± 5 profiles/mm^2^; **p < 0.01). Indeed, even in chronic disease stages, the number of axons undergoing Wallerian degeneration was significantly lower in PPWM areas of chronic inactive lesions (1.4 ± 0.6 profiles/mm^2^) compared to PPWM of active (6.8 ± 1.3 profiles/mm^2^; **p < 0.01), and chronic active lesions (5.1 ± 0.9 profiles/mm^2^; *p = 0.03). Our findings in brains of patients with multiple sclerosis indicate a high incidence of Wallerian degeneration in early stages of multiple sclerosis, which may be related to axonal transections in focal demyelinated lesions.

### No difference in MOG_35–55_ EAE course and severity between *Wld*^*S*^ and WT mice

**Fig. 2:**
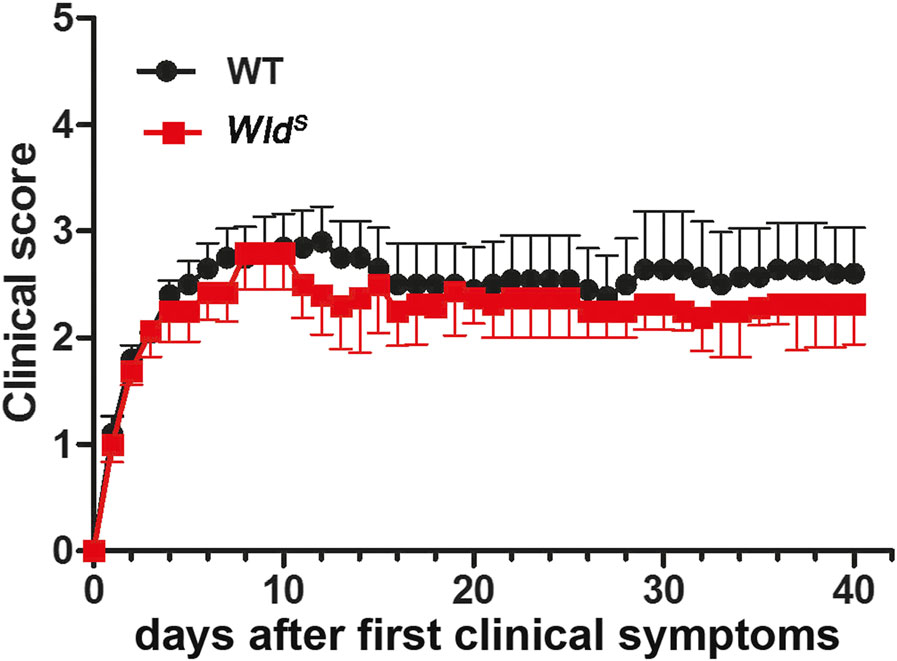
The *Wld* ^*S*^ mutation does not influence the clinical severity of EAE. EAE severity in *Wld* ^*S*^ (*n* = 18) and WT (*n* = 19) mice was similar in both the acute and chronic disease stage. Mean disease score ± SD during 40 days after disease onset are shown. The graph contains scores from three independent experiments. The days after development of the first clinical symptoms are plotted on the *x*-axis

To assess the impact of the *Wld* ^*S*^ fusion gene on inflammatory disease severity, MOG_35–55_ EAE was induced in *Wld* ^*S*^ mutant and WT C57BL/6 mice. No statistically significant difference in disease severity, i.e., mean disease score ± SD (standard deviation) was observed between *Wld* ^*S*^ and WT mice on specifically testing the acute (day 20, *p* = 0.94) and chronic (day 40, *p* = 0.85) disease phase (Fig. 2) [26]. Also, the mean maximal cumulative score 40 days after disease onset was comparable in WT (2.5 ± 0.32) and *Wld* ^*S*^ (2.31 ± 0.28) mice. A qualitative histopathological analysis revealed an increase in lesion sizes from day 20 to day 40 after disease onset in both experimental groups. Experiments were repeated at least three times, and consistent results were obtained.

### Analysis of inflammation and demyelination in MOG_35–55_-induced EAE in *Wld*^*S*^ and WT mice

**Fig. 3:**
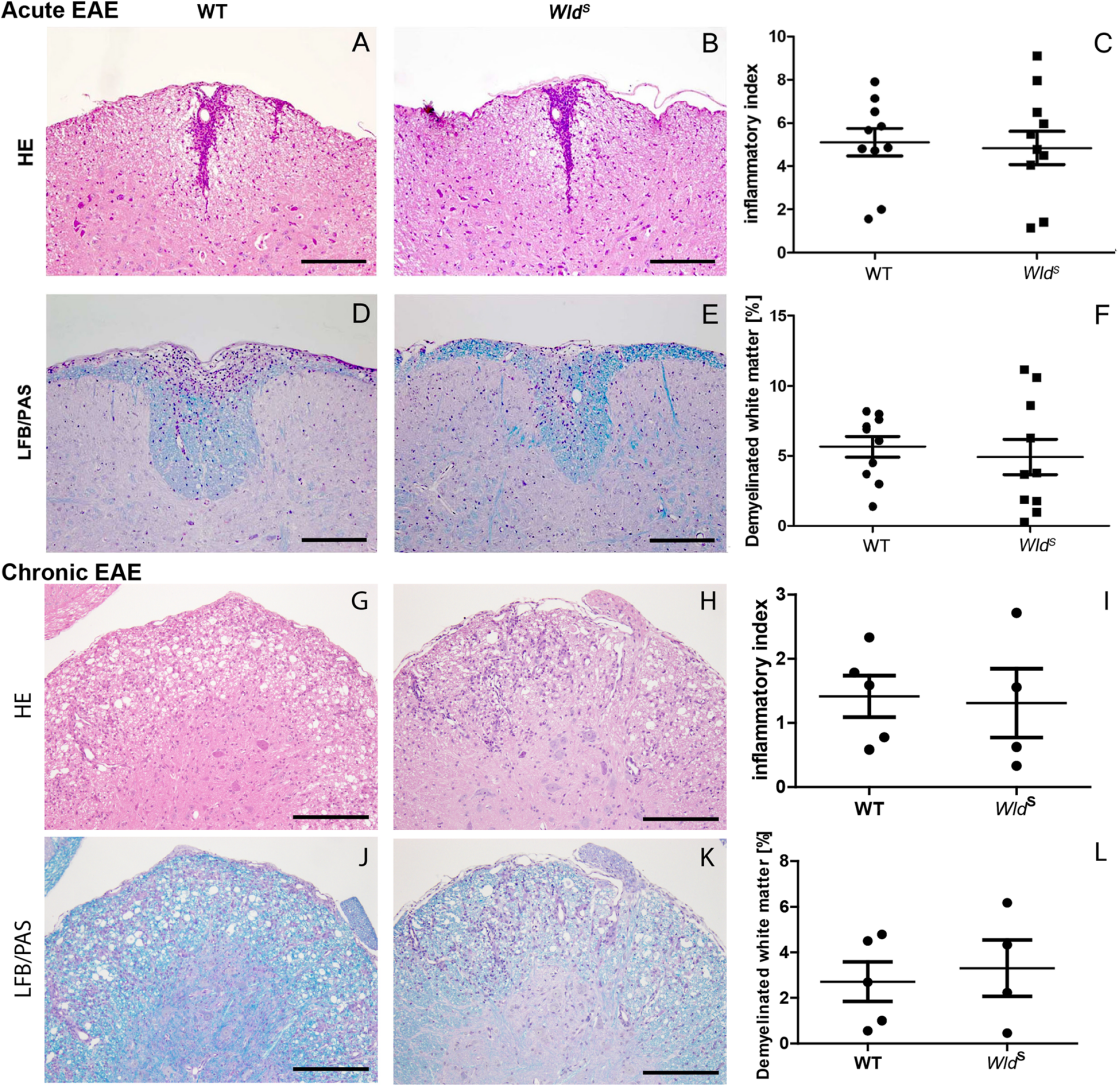
Similar extent of inflammation and demyelination in *Wld* ^*S*^ and WT mice in acute and chronic MOG_35–55_-EAE. The mean number of inflammatory infiltrates per spinal cord (SC) cross section (H&E; **a**–**c**; **g**–**i**), and the percentage of demyelinated white matter (LFB/PAS; **d**–**f**; **j**–**l**) did not differ between *Wld* ^*S*^ and WT mice in the acute as well as the chronic disease stage (d20 and d40). *Error bars*=SEM. *Scale bars*=(**a**, **b**, **d**, and **e**) 100 μm; (**g**, **h**, **j**, and **k**) 200 μm

Differences in lesion acuteness and extent of inflammation and demyelination might obscure differences in the extent of axonal vulnerability between the two mouse strains. We thus examined the number of perivascular and subpial inflammatory infiltrates per SC cross section (inflammatory index) and the percentage of demyelinated white matter in *Wld* ^*S*^ vs. WT mice with acute EAE (Fig. 3a–f). The mean inflammatory index was 4.8 ± 0.8 in *Wld* ^*S*^ and 4.9 ± 0.7 in WT mice (Fig. 3c); the mean demyelinated white matter area was 5.4 ± 1.3% in *Wld* ^*S*^ and 5.7 ± 0.7% in WT mice (Fig. 3f). Inflammatory index and demyelination after immunization with MOG_35–55_ in *Wld* ^*S*^ and WT mice were thus virtually the same, making a direct comparison of axonal pathology between the two mouse strains possible [27]. To ensure that lesions with similar inflammatory demyelinating activity were examined for axonal damage and loss, IHC for the early macrophage activation antigen S100A9 was performed, and all animals were found to have active lesions with recent monocyte invasion [28]. Also, morphometry on H&E and LFB/PAS stained sections of *Wld* ^*S*^ and WT mice with chronic EAE revealed no significant differences with regard to inflammation (mean inflammatory index *Wld* ^*S*^; 1.3 ± 0.5 and WT; 1.4 ± 0.3 inflammatory infiltrates per spinal cord cross section) or demyelination (mean demyelinated white matter *Wld* ^*S*^; 3.3 ± 1.2% and WT; 2.7 ± 0.9%) between the strains (Fig. 3g-l). However, a significant decrease in the extent of inflammation and a trend towards reduced demyelination from day 20 to day 40 after disease onset was observed for both experimental groups (two-way ANOVA; inflammatory index, *F* (1, 25), *p* = 0.0002); demyelination, *F* (1, 25), *p* = 0.0519).

### Similar amount of acute axonal damage in *Wld*^*S*^ and WT EAE mice

**Fig. 4:**
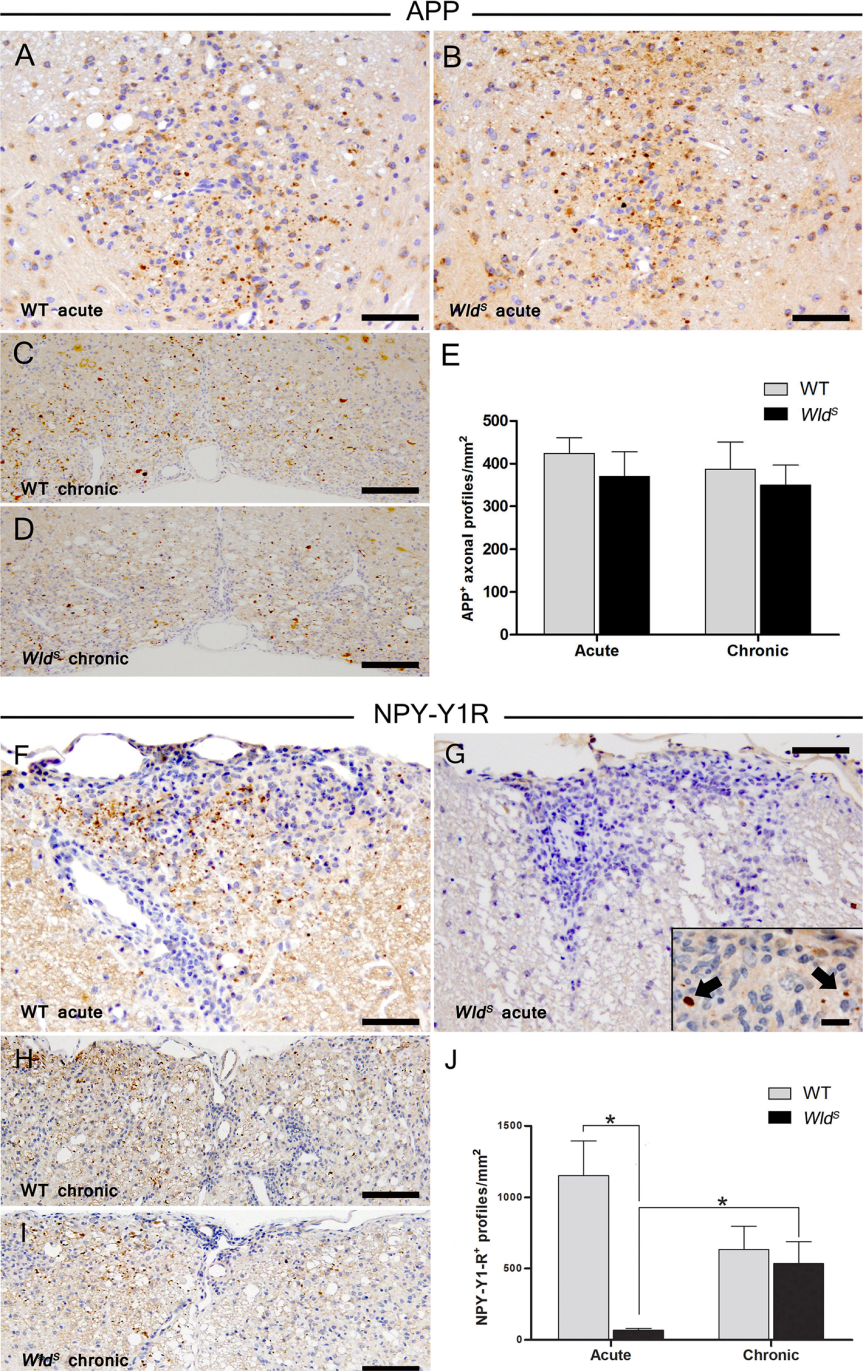
Similar extent of acute axonal damage but markedly reduced Wallerian degeneration in *Wld* ^*S*^ mice with acute EAE. Comparable densities of APP^+^ axonal profiles were found in spinal EAE lesions of *Wld* ^*S*^ and WT mice in both the acute and chronic disease stage (**a**–**e**). In contrast, significantly fewer NPY-Y1R^+^ axonal profiles were observed in *Wld* ^*S*^ as compared to WT mice in acute EAE (**f**–**g** and **j**). In the chronic stage, the densities of NPY-Y1R-immunoreactive axonal profiles were comparable between *Wld* ^*S*^ and WT mice (**h**–**j**). *Error bars*=SEM, *p < 0.05, *n* = 15 animals per group. *Scale bars*=(**a**–**b**) 100 μm; (*inset* **b**) 50 μm; (**c**–**d**) 200 μm; (**f**–**g**) 150 μm; (**h**–**i**) 200 μm

The clinical deficit in an acute inflammatory demyelinating disease may not reflect the extent of structural axonal damage due to the effects of cytokines and edema on ion homeostasis and axonal conduction. In fact, elevated cytokine levels in the CNS could affect neuroaxonal function and may at least be partly responsible for the clinical manifestations during acute exacerbation of multiple sclerosis and EAE [29, 30]. Therefore, to identify the extent of acute axonal damage in *Wld* ^*S*^ mutant and WT mice, we applied antibodies to APP (Fig. 4a–d) to visualize axons with impaired axonal transport. In the acute as well as chronic disease stage, the densities of APP^+^ axonal profiles in inflammatory demyelinated lesions did not differ between *Wld* ^*S*^ and WT mice [*Wld* ^*S*^; 370 ± 58, WT; 424 ± 37 APP^+^ profiles/mm^2^ (acute, *p* = 0.46); *Wld* ^*S*^; 350 ± 47, WT; 387 ± 64 APP^+^ profiles/mm^2^ (chronic, *p* = 0.79)], thus indicating that axons are damaged to a similar degree in both mouse strains, irrespective of the genotype and disease phase (Fig. 4e).

### Less Wallerian degeneration in the acute stage of EAE in *Wld*^*S*^ mutant mice

As characterized previously [31, 32], the NPY-Y1R antiserum recognized elongated and ovoid axonal structures in *Wld* ^*S*^ and WT EAE mice (Fig. 4f–g). In acute disease, a mean of 67 ± 13 NPY-Y1R^+^ axonal profiles/mm^2^ in *Wld* ^*S*^ mutant mice and 1153 ± 241 NPY-Y1R^+^ axonal profiles/mm^2^ in WT mice were detected (Fig. 4f–g and j), indicating markedly reduced Wallerian degeneration in *Wld* ^*S*^ mice in the acute phase of the disease (*p = 0.02). In contrast, no significant difference in Wallerian degeneration was observed in the chronic disease phase; 535 ± 154 NPY-Y1R^+^ axonal profiles per square millimeter were detected in *Wld* ^*S*^ mice and 633 ± 164 per mm^2^ in *Wld* ^*S*^ mice (*p* = 0.69) (Fig. 4h–j). Intriguingly, a significant increase in the densities of NPY-Y1R^+^ axonal profiles was observed in chronic *Wld* ^*S*^ EAE mice as compared to the acute stage (*p = 0.03). This implies that axon degenerative mechanisms in *Wld* ^*S*^ mice are averted primarily during the acute stage of an autoimmune inflammatory demyelinating attack.

### NPY-Y1R immunoreactivity specifically identifies Wallerian degeneration in EAE and stroke

**Fig. 5:**
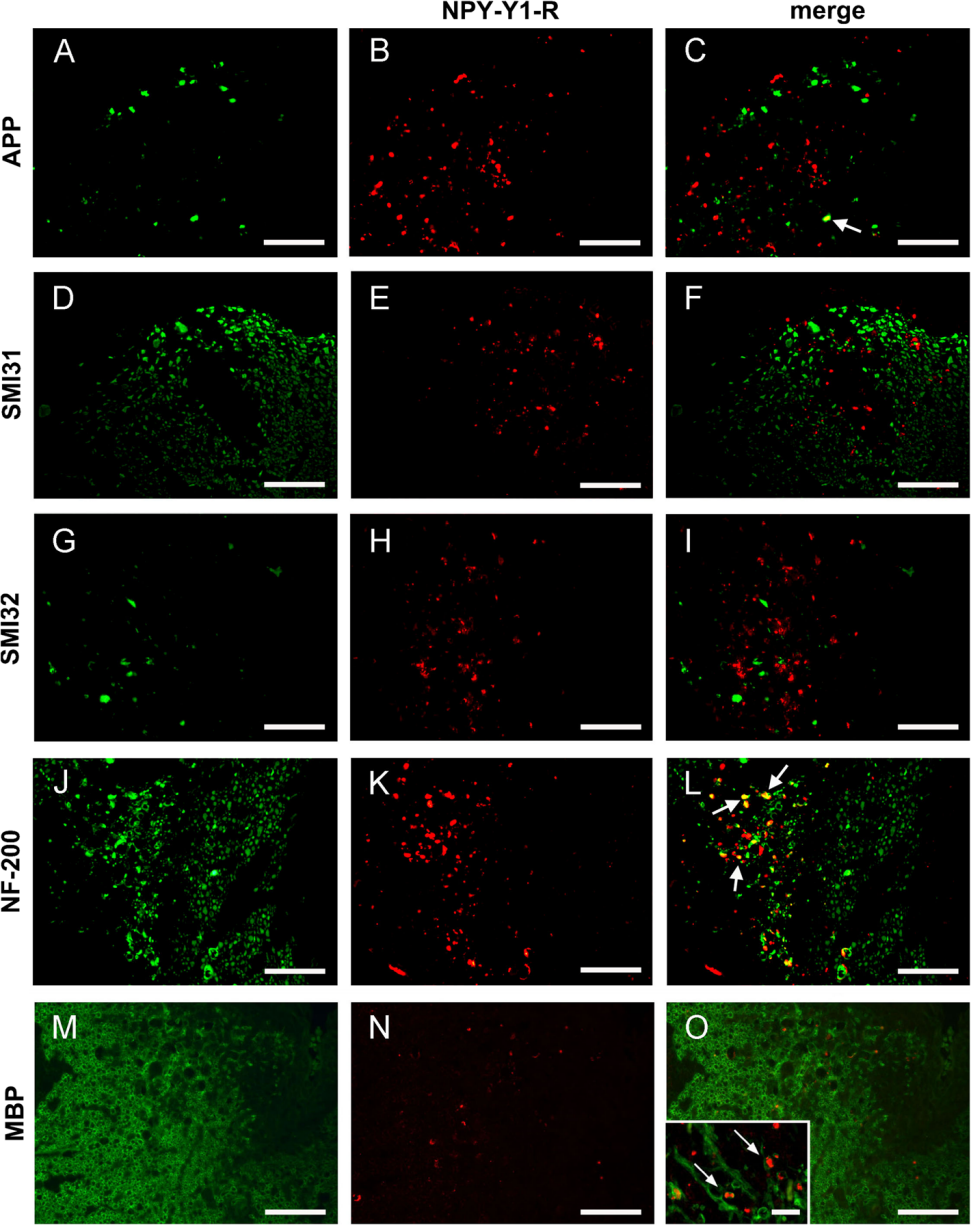
NPY-Y1R does not co-localize with markers of axonal transport disturbance, neurofilament dephosphorylation or myelin proteins in EAE lesions. Immunofluorescence double labeling performed on EAE lesions in WT mice revealed only rare co-localization of NPY-Y1R (*red*) and early axonal transport deficits indicated by APP accumulation (*green*) (**a**–**c**, *arrow*). NPY-Y1R immunoreactivity also did not co-localize with phosphorylated neurofilaments (NF) of healthy axons (SMI31, *green*) (**d**–**f**) or de-, resp. non-phosphorylated NF predominantly found in damaged axons (SMI32, *green*) (**g**–**i**), but partly with the NF high molecular weight (NF-200, *green*) subunit (**j**–**l**, *arrows*). Double IHC of NPY-Y1R (**n**, *red*) with MBP (**m**, *green*) did not reveal any co-localization (**o**, *inset*). *Inset* in (**o**) shows NPY-Y1R^+^ axons, in part enwrapped by myelin, at high magnification (*arrows*). *Scale bars*=(**a**–**o**) 200 μm; (*inset* **o**) 10 μm

To examine whether the Wallerian degeneration-induced expression of NPY-Y1R is associated with degenerating axonal structures, immunofluorescence double labelings were performed in mouse EAE lesions (Fig. 5). We observed only rarely a co-localization of NPY-Y1R immunoreactivity with accumulated APP in axons (Fig. 5a–c). Also, NPY-Y1R expression did not co-localize with phosphorylated neurofilaments (NFs) of healthy axons detected by the SMI31 antibody (Fig. 5d–f), dephosphorylated NFs of damaged axons detected by the SMI32 antibody (Fig. 5g–i), and the low-molecular-weight NF-68 (Additional file 1). Hypophosphorylated NFs found in normal axons recognized by the SMI35 antibody were only occasionally co-localized with NPY-Y1R^+^ axons (Additional file 1). However, substantial co-localization of NPY-Y1R was observed with the high-molecular-weight NF-200, detected by the N52 antibody (Fig. 5j–l, Table 2). Besides axonal degeneration, Wallerian degeneration is characterized by disintegration of myelin with the formation of the pathognomonic myelin ovoids [33]. Hence, to assess whether the antigen detected by the anti-NPY-Y1R antiserum is associated with myelin ovoids, we performed fluorescent double IHC with antibodies against myelin basic protein (MBP), myelin proteolipid protein (PLP), MOG, myelin-associated glycoprotein (MAG), and 2′,3′-Cyclic-nucleotide 3′-phosphodiesterase (CNPase) in spinal EAE lesions from WT mice (Fig. 5m–o, Additional file 2). NPY-Y1R did not co-localize with any of the myelin proteins. In addition, to test whether this spatial co-localization pattern is also observed in areas of Wallerian degeneration after stroke, human ischemic brain lesions were double-labeled for NPY-Y1R and axonal markers (data not shown). In line with our findings in mouse EAE, co-localization of NPY-Y1R and NF-200 was observed, but not of NPY-Y1R and APP or SMI32.

### NPY-Y1R^+^ axonal structures are enwrapped by myelin in early experimental sciatic nerve transection

Wallerian degeneration after mouse sciatic nerve axotomy features a rapid degeneration of axons during the first 6 days after injury [34, 35]. Double immunofluorescence on mouse sciatic nerve 6 days after transection showed significantly more elongated and ovoid NPY-Y1R^+^ axonal structures enwrapped by MBP^+^ myelin sheaths than degenerated axons lacking any myelin (Additional file 3). This further supports that NPY-Y1R expression is present on axonal structures, but not myelin, in both the CNS and PNS, thus identifying comparatively early stages of Wallerian degeneration.

### Axonal loss is similar in *Wld*^*S*^ and WT mice in the chronic disease phase

**Fig. 6:**
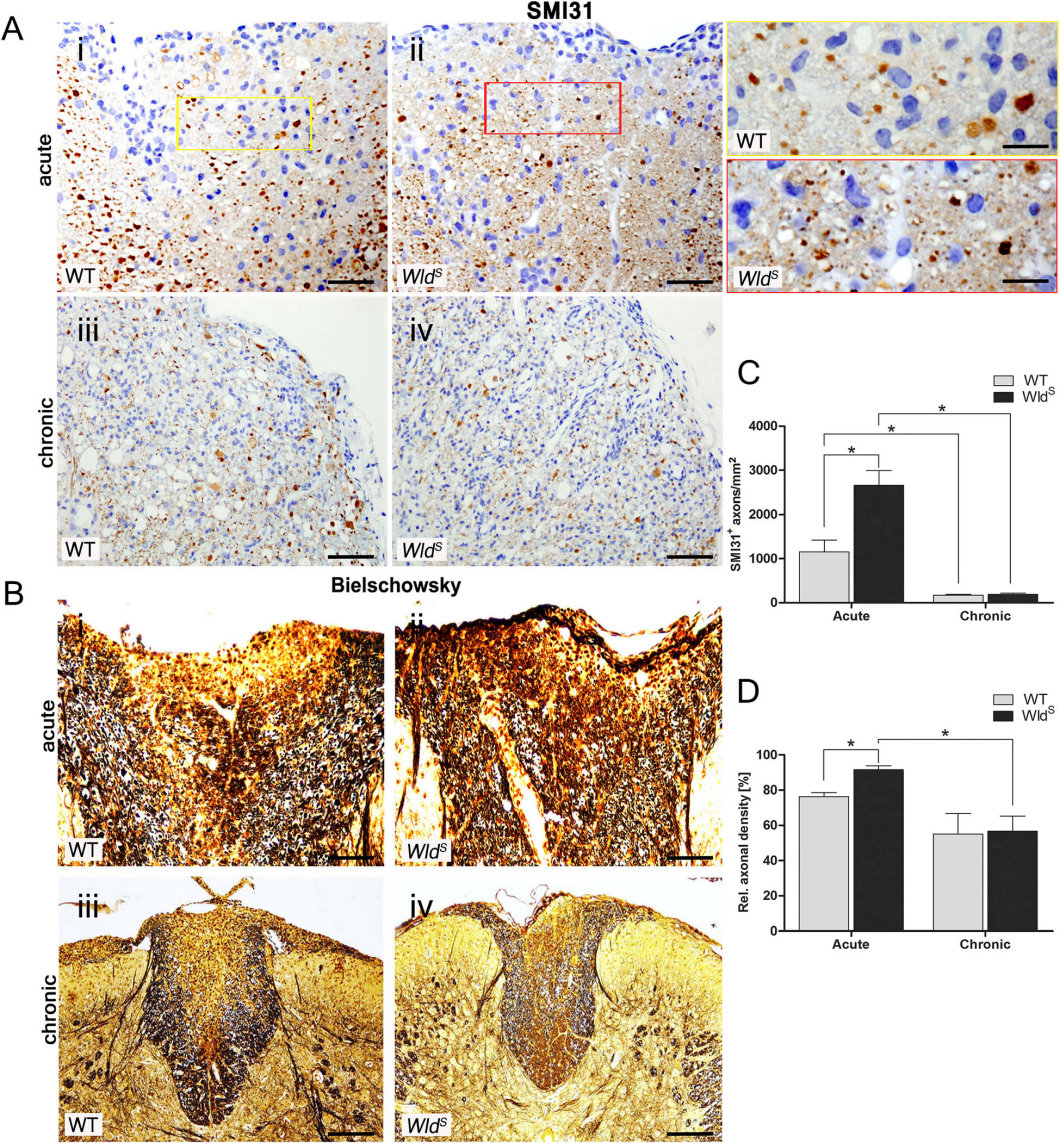
Less axonal loss in the acute disease phase in *Wld* ^*S*^ mutant mice. SC cross sections of *Wld* ^*S*^ mutant and WT mice with EAE were stained with anti-SMI31 antibody (**a**) and Bielschowsky’s silver impregnation (**b**) showing axonal loss in white matter lesions 20 days (acute) and 40 days (chronic) after disease onset. Quantification of SMI31^+^ phosphorylated axons revealed that the densities of intact healthy axons were significantly higher in *Wld* ^*S*^ mice as compared to WT mice in acute stage disease (**a** *i–ii*, **c**). However, similarly dense SMI31^+^ profiles were observed in the chronic stage (**a** *iii*–*iv*; lateral funiculus, **c**). Densities of SMI31^+^ profiles significantly decreased in both *Wld* ^*S*^ and WT mice from the acute to the chronic disease stage (**a, c**). Similarly, Bielschowsky’s silver impregnation displayed significantly higher relative axonal densities in *Wld* ^*S*^ mice only in the acute stage (**b** *i*–*ii*, **d**); again, axonal loss in *Wld* ^*S*^ mice was significantly more pronounced in the chronic than in the acute disease stage (**b**, **d**). *Yellow and red boxes* in (**a** *i*–*ii*) are higher magnifications of lesion areas with SMI31 immunostaining in acute disease. *Error bars*=SEM, *n* = 15 animals per group, *p < 0.05. *Scale bars*=(**a** *i*–*ii*) 100 μm; (**a** *iii*–*iv*, **b** *i*–*ii*) 150 μm; (**b** *iii*–*iv*) 200 μm; (*insets* **a** *i*–*ii*) 25 μm

We hypothesized that the lower numbers of axons undergoing Wallerian degeneration in the early disease stage might lead to less net axonal loss in established EAE lesions of *Wld* ^*S*^ mice. Healthy SMI31^+^ axons as well as relative axonal densities (Bielschowsky silver impregnation) were determined in EAE lesions (Fig. 6). In the acute stage, the number of healthy SMI31^+^ axons was significantly higher in *Wld* ^*S*^ compared to WT lesions (*Wld* ^*S*^; 2658 ± 335, WT; 1156 ± 265 SMI31^+^ profiles/mm^2^; *p = 0.02), whereas the counts were very similar in the chronic stage (*Wld* ^*S*^; 193 ± 21, WT; 173 ± 14 SMI31^+^ profiles/mm^2^; *p* = 0.39) (Fig. 6a and c). Furthermore, the densities of healthy axons in both genotypes were significantly higher in the acute compared to the chronic stage (*Wld* ^*S*^; *p = 0.03; WT; *p = 0.01). Importantly, axons after an inflammatory attack may become dephosphorylated but do not necessarily vanish. Therefore, we next quantified axons in defined lesion areas after Bielschowsky silver impregnation (Fig. 6b and d). Relative axonal densities were significantly higher in *Wld* ^*S*^ mice than WT mice in the acute stage (*Wld* ^*S*^; 91.52 ± 2.3%, WT; 76.34 ± 2.3%; *p = 0.04) but virtually identical in the chronic stage (*Wld* ^*S*^; 56.8 ± 8.4%, WT; 55.1 ± 11.6%; *p* = 0.93). Moreover, comparable with the SMI31^+^ counts, relative axonal densities of silver impregnated axons in *Wld* ^*S*^ mice were significantly lower in the chronic than in the acute stage (*p = 0.03). This clearly indicates that the *Wld* ^*S*^ mutation did not protect against long-term inflammatory axonal loss. Hence, consistent with other CNS lesion paradigms tested [36–38], our results suggest that also in inflammatory demyelination, Wallerian degeneration is merely slowed down or delayed in *Wld* ^*S*^ mice.

### Markers for axonal regeneration and synaptic plasticity are not co-expressed with NPY-Y1R in degenerating axons

De- and regenerative processes in axons may be closely related. We thus tested whether markers putatively associated with axonal regeneration are co-expressed with NPY-Y1R. Growth-associated protein 43 (GAP43) and synaptophysin (Syn) are synaptic proteins that are associated with axonal sprouting and synaptogenesis in various neurodegenerative diseases and may accumulate in regenerating neurons and axons. Therefore, expression of GAP43 and Syn in damaged axons could reflect a regenerative attempt [24]. However, immunofluorescence double labelings on WT EAE spinal lesions did not reveal any co-localizations of NPY-Y1R with GAP43 (Additional file 4 A-C) or Syn (Additional file 4 D-F).

## Discussion

The extent of axonal loss is the most important predictor of permanent clinical deficits in multiple sclerosis [39–41]. Wallerian degeneration of axons transected in focal inflammatory demyelinated lesions contributes to the loss of neural structures. Here, we demonstrate a clear relationship between lesion demyelinating activity and Wallerian degeneration in non-demyelinated multiple sclerosis white matter. Furthermore, we find that the extent of Wallerian degeneration is most extensive in early disease stages supporting the interrelation between inflammation and neurodegeneration in multiple sclerosis [24, 42, 43]. In addition, in the present study, we hypothesized that *Wld* ^*S*^ mutant mice would present with less axonal damage and reduced disability in MOG_35–55_ EAE compared to WT mice. However, surprisingly, *Wld* ^*S*^ and WT mice showed a similar disease course, and the degree of inflammation, demyelination, and acute axonal damage was comparable in the early as well as in the more advanced disease stage. Immunohistochemical staining with the anti-NPY-Y1R antibody demonstrated abundant Wallerian degeneration in WT mice in the early and chronic disease stage but identified only very few axons undergoing Wallerian degeneration in *Wld* ^*S*^ mice in the acute disease stage. Furthermore, the net axonal loss was comparable in the chronic disease stage in both genotypes. Our data thus indicate that the *Wld* ^*S*^ gene markedly delays Wallerian degeneration after inflammatory axonal damage but does not ameliorate disability resulting from EAE or cumulative axon loss.

Previous studies investigating axotomy-induced CNS Wallerian degeneration proposed three distinct phases: first, the sudden fragmentation (after 10–20 min) of both the distal and proximal axon close to the site of injury; second, the slow axonal retraction resulting in bulb formation at the axon ends with the distal stump remaining anatomically integrated and functional, and third, a proximal stable fragment while the distal fragment undergoes secondary degeneration, i.e., swelling, connection thinning, and rapid granularization [20, 44–47]. Convincing evidence for the distinct molecular basis of these processes was recently obtained in studies performed in *Wld* ^*S*^ mutant mice. Kerschensteiner and colleagues could show that the *Wld* ^*S*^ mutation prevented the onset of sudden fragmentation in transected CNS axons [20]. Also, expression of the *Wld* ^*S*^ gene remarkably protracted the slow retraction phase in which the axon distal to the transection site remains morphologically intact and retains its physiologic function [34, 38, 48, 49].

Modulation of ion channels expressed in axons under inflammatory demyelinating conditions was shown to amend axonal degeneration [50–52]. Interestingly, it has been demonstrated that Wallerian degeneration of the distal stump is a Ca^2+^-mediated process [53, 54]. The rise in intra-axonal ROS production in the distal stump after transection is diminished in the *Wld* ^*S*^ mutation, which thus may entail preserved mitochondrial function and energy supply [55]. Hence, the fast fragmentation and slow retraction, but not the late rapid granularization, are ROS dependent. This is in agreement with the notion that the delay in axonal degeneration observed in *Wld* ^*S*^ axons is only transient. Mitochondrial dysfunction, energy failure, and ensuing Ca^2+^ overload have been implicated as the final common pathways in inflammatory axonal damage, leading to the activation of intra-axonal proteases, cytoskeletal degradation, and axonal degeneration and loss [56, 57]. Therefore, the *Wld* ^*S*^ mutation might be well suited to protect axons in an inflammatory demyelinating milieu, even beyond its well-described effect on the classical axonal self-destruction after transection. In our study, however, the densities of acutely damaged axons in inflammatory lesions were similar in *Wld* ^*S*^ and WT mice, in both the acute and chronic disease stages, suggesting that the inflammatory insult surpassed the axonoprotective capacities of the *Wld* ^*S*^ phenotype.

Our data show that the occurrence of Wallerian degeneration in multiple sclerosis is related to the density of axons with transport deficits observed in the tissue: Wallerian degeneration is most abundant in the periplaque white matter of patients early in the disease course harboring macrophage-rich focal lesions with ongoing myelin degradation. To the contrary, only few axons undergoing Wallerian degeneration were observed in late-stage patients with predominantly chronic inactive lesions. Noteworthy and as reported previously, chronic active lesions represent an area of ongoing demyelination and focal axonal degeneration [2, 14, 58]. In line, elevated levels of axons undergoing Wallerian degeneration were detected in the corresponding periplaque and normal-appearing white matter. Although our work cannot definitely exclude the presence of focal axonal degeneration in non-lesional areas, it clearly shows that the extent of Wallerian degeneration is related to focal demyelinating pathology. Furthermore, our work highlights the relevance of axonoprotective treatments, also in patients with more advanced disease, where smoldering lesions are most common.

Coleman and colleagues [59, 60] observed that the *Wld* ^*S*^ mutation also inhibits degenerative axonal swellings in gracile axonal dystrophy (*gad*) mice, a mutant mouse strain characterized by a “dying-back” type axonal degeneration and the formation of axonal spheroids. In contrast, we studied axonal pathology under inflammatory demyelinating conditions. NOS and ROS released by activated macrophages have been implicated in acute axonal transport disturbance, even in myelinated axons, and may contribute to mitochondrial damage and Ca^2+^ overload, leading to a vicious cycle of energy failure and cytoskeletal degradation [10]. Our results suggest that the intra-axonal protective mechanisms operating in *Wld* ^*S*^ mice, e.g., antioxidation and increased Ca^2+^ buffering capacity, are not sufficient to prevent or diminish axonal swellings during a myelin-specific T cell-mediated autoimmune attack. Prior studies showed that the *Wld* ^*S*^ protection mechanism is intrinsic to the axon [47, 61, 62]. However, recently, an upregulation of CD200 was observed in *Wld* ^*S*^ mice, which may play a critical role in reducing microglia-mediated neuroaxonal damage in the CNS [63–65]. Taken together, our results indicate that in inflammatory demyelination, axonal transport deficits and cumulative loss of axons are not restrained in *Wld* ^*S*^ mice.

Irrespective of disease-related or mechanical transection of axons, the common downstream mechanisms of axonal degeneration include energy failure, increased Ca^2+^ influx and activation of calpains, resulting in the degradation of axon components [66–68]. As early as in the 1980s, it has been shown that NPY along with its Y1 receptor modulates increased Ca^2+^ concentrations in cultured dorsal root ganglion (DRG) neurons [69, 70]. NPY is widely distributed in the CNS and PNS, albeit NPY and its receptor Y1 are not expressed in the same population of neurons. However, both are upregulated in axonal processes of DRG neurons after nerve injury in rodents [71–74]. It seems therefore possible that NPY-Y1R plays a role in limiting the early destructive processes in damaged axons. In this study, we observed an important delay in NPY-Y1R expression in axons of *Wld* ^*S*^ mice with EAE, which is in line with the reduced Ca^2+^ influx in transected *Wld* ^*S*^ axons. Intriguingly, the increased Ca^2+^ buffering capacity of *Wld* ^*S*^ mitochondria shows a gradual decrease over time in the distal stump [53]. Hence, the intra-axonal protective mechanisms appear to decrease with time after transection in *Wld* ^*S*^ axons. Indeed, we observed substantially decreased immunoreactivity of NPY-Y1R in inflammatory SC lesions of *Wld* ^*S*^ mice in the acute stage as compared to the chronic stage. Our results thus support the use of anti-NPY-Y1R antibodies as a bona fide marker for Wallerian degeneration.

## Conclusions

The present study provides evidence that axonal degeneration in multiple sclerosis is closely related to the occurrence of focal axonal transport disturbances, to focal demyelination and thus to phagocyte activation. Our experimental data indicate that the *Wld* ^*S*^ phenotype does not prevent axonal swellings in inflammatory demyelination and fails to provide sufficient long-term protection of CNS axons in a model of multiple sclerosis. Although we observed an important delay in Wallerian degeneration, this did not result in disease attenuation or a net rescue of axons in *Wld* ^*S*^ mice. Hence, in our model, a delay in Wallerian degeneration does not correlate with immediate clinical amelioration of inflammatory axon damage. Further molecular studies into the axonoprotective mechanisms elicited in the *Wld* ^*S*^ mutant are required to find a way to impede axonal degeneration and eventually restore axonal integrity and function.

## Additional files

Additional file 1: NPY-Y1R+ axon undergoing Wallerian degeneration do not co-localize with hypophosphorylated and low-molecular-weight NF in EAE. Double-labeling fluorescent IHC reveals that NPY-Y1R^+^ degenerating axons are only rarely labeled with antibodies recognizing hypophosphorylated NF (SMI35) (A-C). No colocalization is observed with the 68 kDa low-molecular-weight NF (D-F) in WT EAE lesional and perilesional tissue. *Scale bars*=(A-F) 100 μm. (JPG 343 kb)
Additional file 2: Axons undergoing Wallerian degeneration are at least in part myelinated in EAE lesions. No co-localization of NPY-Y1R immunoreactivity with myelin proteins, i.e., PLP (A-C), MOG (D-F), MAG (G-I), and CNPase (J-L) was observed by fluorescence double IHC in WT EAE mice, which further confirms that the antiserum against NPY-Y1R applied does not detect an antigen situated within the myelin sheath or myelin ovoids. Insets in (A-C) represent NPY-Y1R^+^ degenerating fiber(s) in largely intact myelinated tracts, as determined by anti-PLP IHC. *Scale bars*=(A-L) 200 μm; (insets A-C) 10 μm. (JPG 673 kb)
Additional file 3: NPY-Y1R IHC labels myelin ovoids typical of Wallerian degeneration in mouse sciatic nerve transection. Elongated, beaded NPY-Y1R^+^ (red, A) axonal structures surrounded by MBP^+^ myelin sheaths (green, B) are seen in longitudinal sections of mouse sciatic nerve 6 days after and distal to the transection (C, arrows). Oil-immersion magnification (×1000) revealed that NPY-Y1R^+^ axons were enwrapped with myelin (MBP) indicating myelin ovoid formation, typical of Wallerian degeneration (D-F). (*p* < 0.05; G). *Scale bars*=(A-C) 100 μm; (D-F) 20 μm. (JPG 1484 kb)
Additional file 4: Neuroaxonal regenerative markers are not co-expressed in axons undergoing Wallerian degeneration. Axonal structures immunopositive for GAP43 (A-C) and Synaptophysin (Syn) (D-F) did not co-localize with NPY-Y1R^+^ degenerating axons in WT EAE lesions by immunofluorescent double labeling. Syn expression was mostly limited to gray matter regions of the SC in WT EAE. *Scale bars*=(A-F) 100 μm. (JPG 816 kb)

## Abbreviations

APP: Amyloid precursor protein
EAE: Experimental autoimmune encephalomyelitis
NAWM: Normal-appearing white matter
NPY-Y1R: Neuropeptide Y-Y1 receptor
PPWM: Periplaque white matter
*Wld*^*S*^: Wallerian degeneration slow
