# Supplementary figures and images for: Relationship of acute axonal damage, Wallerian degeneration, and clinical disability in multiple sclerosis
Source: J Neuroinflammation. 2017 Mar 17;14:57. doi: 10.1186/s12974-017-0831-8 (PMC5356322; doi:10.1186/s12974-017-0831-8)

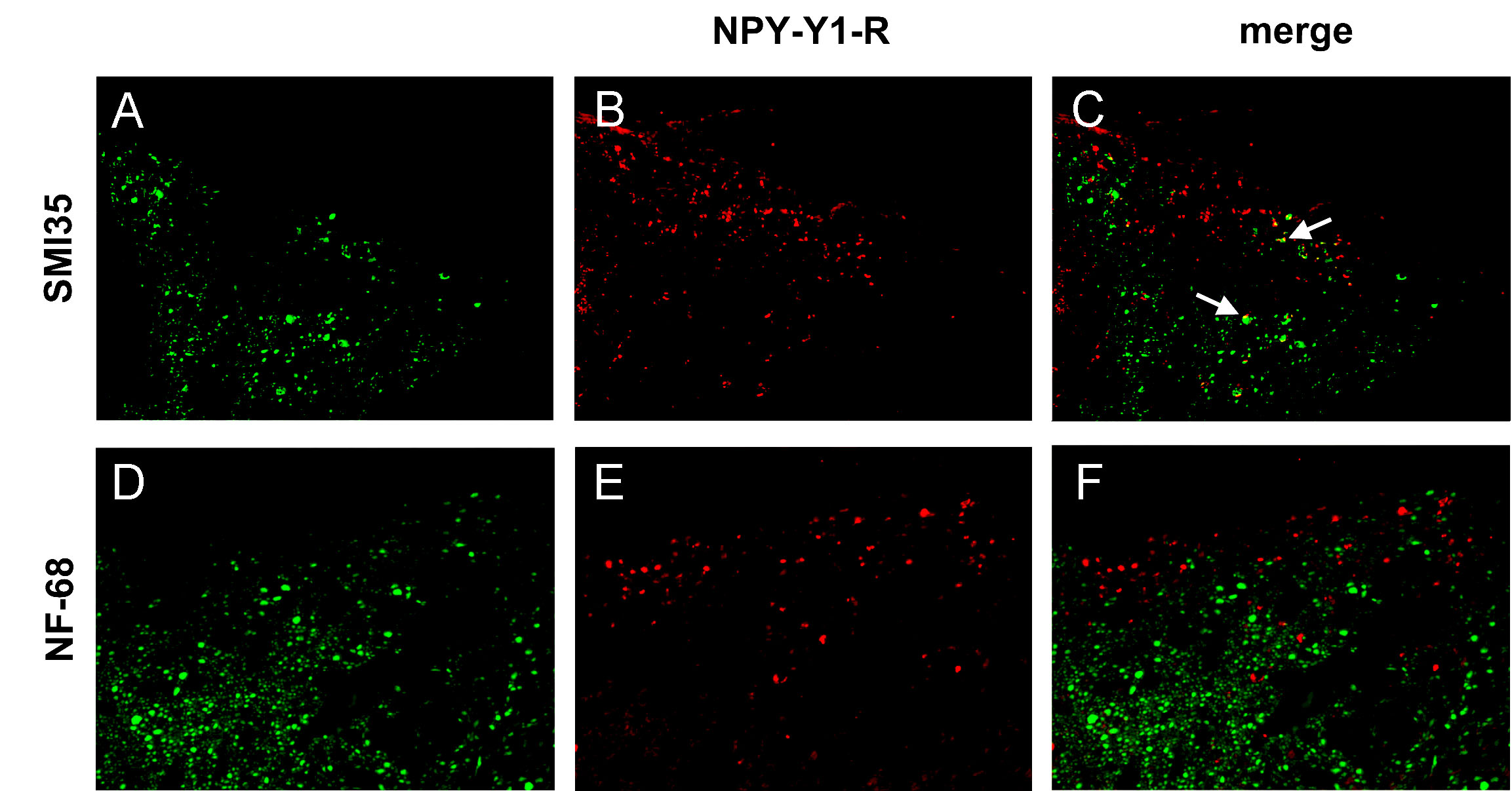

Supplement: Additional file 1: — NPY-Y1R+ axon undergoing Wallerian degeneration do not co-localize with hypophosphorylated and low-molecular-weight NF in EAE. Double-labeling fluorescent IHC reveals that NPY-Y1R+ degenerating axons are only rarely labeled with antibodies recognizing hypophosphorylated NF (SMI35) (A-C). No colocalization is observed with the 68 kDa low-molecular-weight NF (D-F) in WT EAE lesional and perilesional tissue. Scale bars=(A-F) 100 μm. (JPG 343 kb) [file 12974_2017_831_MOESM1_ESM.jpg]

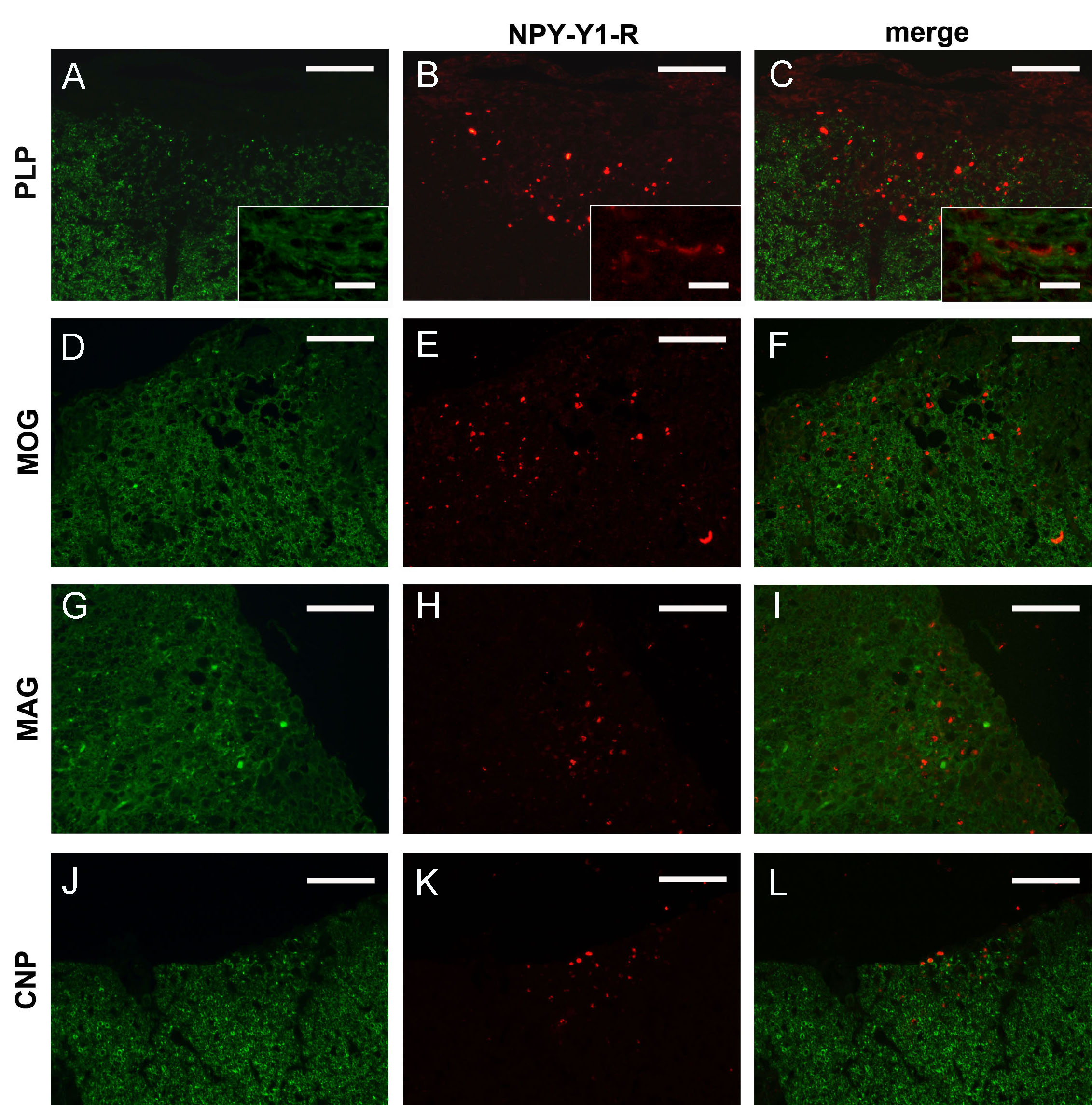

Supplement: Additional file 2: — Axons undergoing Wallerian degeneration are at least in part myelinated in EAE lesions. No co-localization of NPY-Y1R immunoreactivity with myelin proteins, i.e., PLP (A-C), MOG (D-F), MAG (G-I), and CNPase (J-L) was observed by fluorescence double IHC in WT EAE mice, which further confirms that the antiserum against NPY-Y1R applied does not detect an antigen situated within the myelin sheath or myelin ovoids. Insets in (A-C) represent NPY-Y1R+ degenerating fiber(s) in largely intact myelinated tracts, as determined by anti-PLP IHC. Scale bars=(A-L) 200 μm; (insets A-C) 10 μm. (JPG 673 kb) [file 12974_2017_831_MOESM2_ESM.jpg]

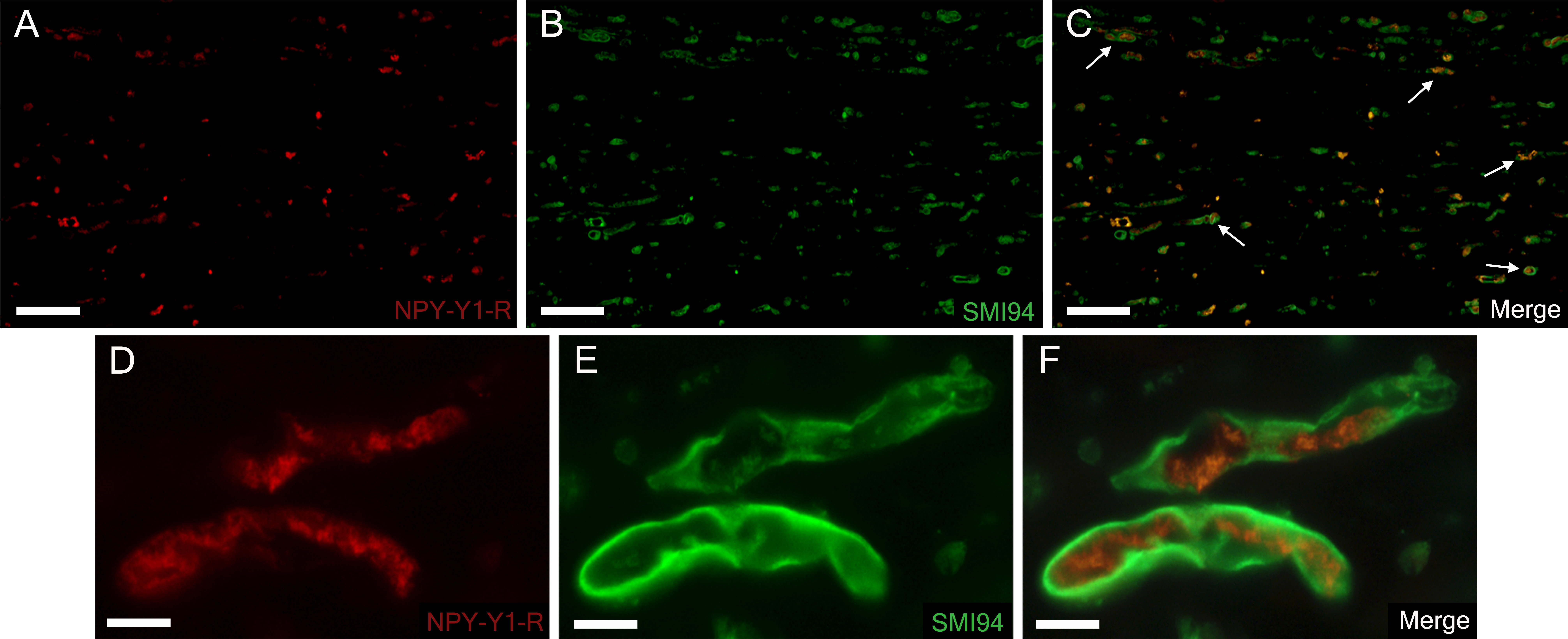

Supplement: Additional file 3: — NPY-Y1R IHC labels myelin ovoids typical of Wallerian degeneration in mouse sciatic nerve transection. Elongated, beaded NPY-Y1R+ (red, A) axonal structures surrounded by MBP+ myelin sheaths (green, B) are seen in longitudinal sections of mouse sciatic nerve 6 days after and distal to the transection (C, arrows). Oil-immersion magnification (×1000) revealed that NPY-Y1R+ axons were enwrapped with myelin (MBP) indicating myelin ovoid formation, typical of Wallerian degeneration (D-F). (p < 0.05; G). Scale bars=(A-C) 100 μm; (D-F) 20 μm. (JPG 1484 kb) [file 12974_2017_831_MOESM3_ESM.jpg]

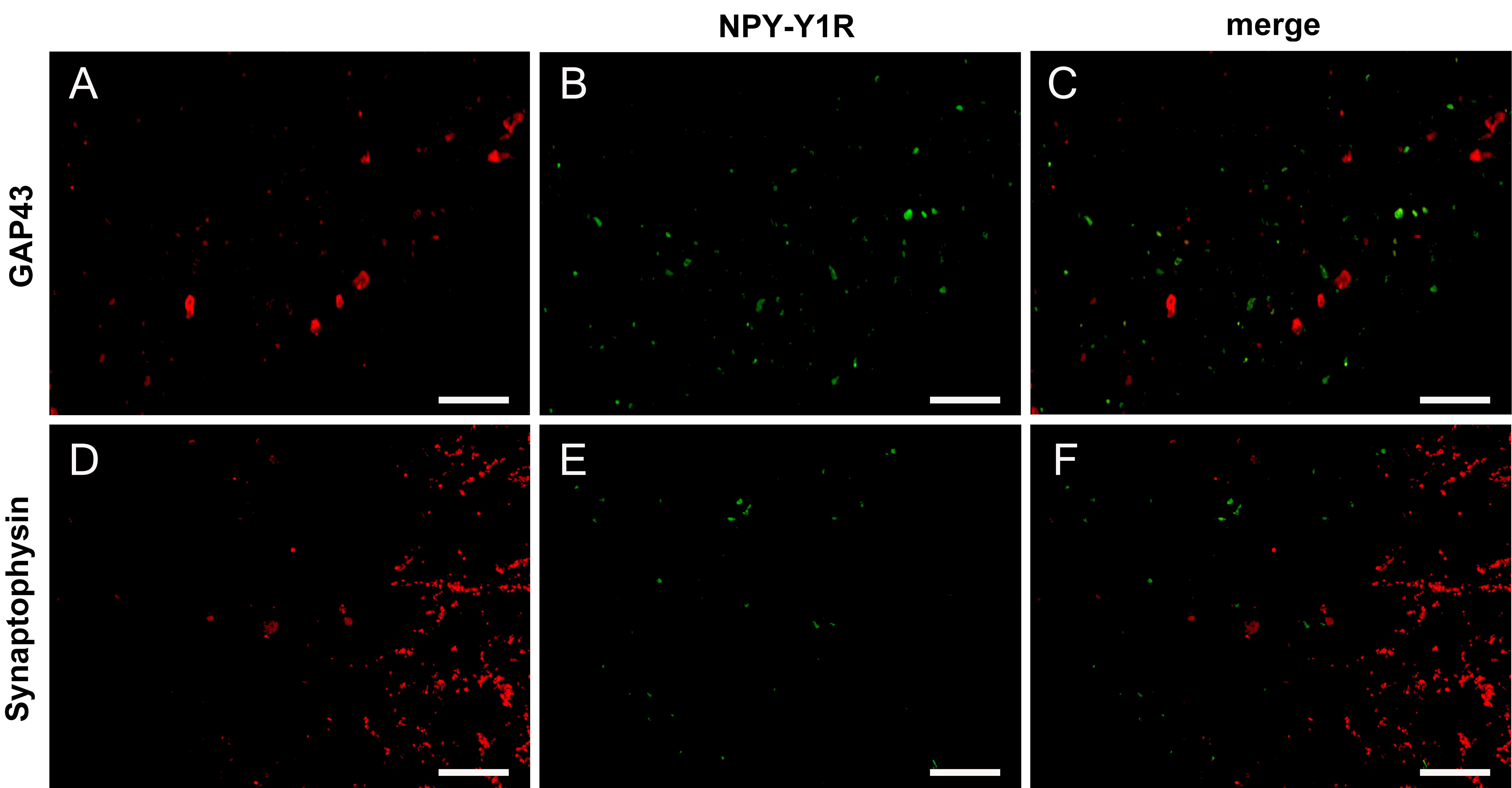

Supplement: Additional file 4: — Neuroaxonal regenerative markers are not co-expressed in axons undergoing Wallerian degeneration. Axonal structures immunopositive for GAP43 (A-C) and Synaptophysin (Syn) (D-F) did not co-localize with NPY-Y1R+ degenerating axons in WT EAE lesions by immunofluorescent double labeling. Syn expression was mostly limited to gray matter regions of the SC in WT EAE. Scale bars=(A-F) 100 μm. (JPG 816 kb) [file 12974_2017_831_MOESM4_ESM.jpg]
